# Supplementary material for: Motivators and barriers to research participation for individuals with cerebral palsy and their families
Source: PLoS One. 2022 Jan 26;17(1):e0262153. doi: 10.1371/journal.pone.0262153 (PMC8791530; doi:10.1371/journal.pone.0262153)
Supplement: S1 Table — *Abbreviations: GMFCS = Gross Motor Function Classification System, di = diplegia, hemi = hemiplegia, quad = quadriplegia. (DOCX) [file pone.0262153.s001.docx]

| **Variable** | **GMFCS Level**  **α = 0.005** | **CP Type**  **α = 0.0083** | **Responder Type**  **α = 0.05** |
| --- | --- | --- | --- |
| ***Additional travel needs*** |  |  |  |
| Time |  | Di > Hemi  Quad > Hemi |  |
| Breathing | Level V > Level IV  Level V > Level I |  |  |
| Transition |  |  | Adults > Parents |
| Seizure | Level V > Level IV  Level V > Level II |  | Parents > Adults |
| Feeding | Level V > Level IV  Level V > Level III  Level V > Level II  Level V > Level I | Quad > Di  Quad > Hemi | Parents > Adults |
| Snacks |  |  | Parents > Adults |
| Medications | Level V > Level IV  Level V > Level III  Level V > Level II  Level V > Level I |  |  |
| Toileting | Level V > Level IV  Level V > Level III  Level V > Level II  Level V > Level I  Level IV > Level II | Quad > Di  Quad > Hemi | Parents > Adults |
| Transportation | Level V > Level II  Level V > Level I  Level IV > Level II  Level IV > Level I  Level III > Level II  Level III > Level I | Quad > Di  Quad > Hemi  Quad > Other  Di > Hemi |  |
| Other |  |  |  |
| ***Transportation*** |  |  |  |
| Drive self |  | Hemi > Di  Hemi > Quad | Parents > Adults |
| Family member drives |  |  |  |
| Public transit |  |  | Adults > Parents |
| Ride service |  |  | Adults > Parents |
| Other |  |  | Adults > Parents |
| ***Locations*** |  |  |  |
| Current clinic |  |  | Parents > Adults |
| New clinic |  | Hemi > Quad |  |
| Park | Level I > Level II  Level I > Level III  Level I > Level IV  Level I > Level V |  | Parents > Adults |
| Lab |  | Hemi > Quad |  |
| School |  |  | Parents > Adults |
| Home |  |  |  |
| Other |  |  |  |
