# Supplementary material for: Motivators and barriers to research participation for individuals with cerebral palsy and their families
Source: PLoS One. 2022 Jan 26;17(1):e0262153. doi: 10.1371/journal.pone.0262153 (PMC8791530; doi:10.1371/journal.pone.0262153)
Supplement: Attachment — Submitted filename: PLOS ONE Response to Reviewers.docx [file pone.0262153.s003.docx]

Reviewer(s)' **Comments to Authors** and Responses

**(1) The manuscript is very good, the essence and concept are very clear and precise. A topic of this research has importance in the motivations and increases the number of respondents in the future research. It will be useful for future researchers in these fields.**

Thank you for your comments and for taking the time to review the manuscript.

**(2) Thank you for the opportunity to review this interesting article. It is a worthwhile contribution to the literature, to understand the facilitators and barriers to people with CP and their families participating in research.**

Thank you for this encouraging comment.

**(3) Excellent introduction, sets up the study well.**

Thank you.

**(4) Methods: Considering the topic, even though opinions were sought, it seems a shame there were no people with CP or family members involved as partners in this research? I see now that three family members seem to be acknowledged. Why are they not authors if they were involved in design and recruitment? Did you discuss interpretation of responses with them?**

We appreciate this thoughtful comment. As we’ve now clarified in the acknowledgements section, the three individuals listed are research colleagues of the authors, and we have rectified the accidental omission those affected by CP in this section. Paragraph 2 of the methods section explains that we sought feedback from six individuals affected by CP on the clarity of survey questions, the completeness of content, and the importance of the survey goals. While these individuals were incredibly generous with their time, there were timing constraints associated with the pandemic that made a more substantial commitment difficult. Furthermore, we did not have funding to appropriately compensate them for larger contributions to the work. In relation to those affected by CP that participated in the survey, we plan to disseminate the results to all individuals who consented and selected the optional element to be informed.

**(5) How were the categories and questions decided upon? Were families involved in this or did they just review for readability?**

The original concept for this study was borne from organic discussions among our research team, so the objectives of the study were mostly established before engaging with stakeholders. However, we did make it clear that we were open to feedback at all levels and the stakeholders who responded focused on the way that questions were asked and how the survey was functioning. We clarified this in paragraph 2 of the methods section.

**(6) How was a scale of 1-100 decided upon?**

We used the REDCap digital version of the visual analog scale (VAS) for three personal interests variables (*research importance*, *value research participation*, *compensation importance*) to easily quantify subjective opinions, similar to the VAS used in pain literature. We’ve updated this in paragraph 4 of the methods section.

**(7) The authors don't seem to have used a checklist such as CHERRIES** [**https://www.ncbi.nlm.nih.gov/pmc/articles/PMC1550605/**](https://urldefense.com/v3/__https://www.ncbi.nlm.nih.gov/pmc/articles/PMC1550605/__;!!Dq0X2DkFhyF93HkjWTBQKhk!GFYVVgGI_ZTWmPy0nxhZv59vD_1Z8Ff6ZG-yKKzdFjZkv6NU-T2ZhlkQ3MEtcxADVzK9OZPqwg_y$)

Thank you for bringing this to our attention. We completed the CHERRIES checklist and uploaded it as a separate document.

**(8) Results: A good number of responses (although response rate not reported). It would be helpful if % were written in Table 1, not just numbers. Motor type refers to spasticity/dyskinesis etc., but here it refers to topography.**

Overall response rate (91.4%) has been clarified in the first paragraph of the Results section. Table 1 has been modified to include the percentage of respondents in addition to the numbers for each demographic. Per the reviewer’s suggestion, “motor type” was changed to “motor topography” to reflect the information more appropriately in this category of Table 1.

**(9) Table 2 is difficult to interpret, especially what the three right hand columns are referring to. This needs further explanation. Is the main finding that they differ or don't differ? We are unable to tell from the table what the difference is (i.e. with GMFCS which GMFCS levels are associated with the variable of interest).**

Thank you for your remarks regarding the clarity of Table 2. We have removed the test statistics and degrees of freedom from each column to make the table more digestible, and we added a column to specify the statistical test used for each variable. Significant results in the three right-hand columns indicate differences between the particular respondent grouping (e.g. GMFCS levels, CP types) and the variable of interest. Please note that supplemental Table S1, which is referenced in the text (second paragraph of the ‘Travel Needs’ Results section), lists the significant pairwise comparisons. This table can be referenced for specific differences in GMFCS levels, etc. for each variable.

**(10) Discussion: There is limited literature supporting or critiquing the findings throughout the discussion. However, there are some very helpful findings from this survey, congratulations to the investigators.**

The reviewer raises a valid point; we are limited by the sparsity of existing literature on this topic. We have done our best to identify and discuss relevant previous work. This is all the more reason to do similar studies to gain a broader understanding of the needs and priorities of the cerebral palsy community.
