# Supplementary material for: Motivators and barriers to research participation for individuals with cerebral palsy and their families
Source: PLoS One. 2022 Jan 26;17(1):e0262153. doi: 10.1371/journal.pone.0262153 (PMC8791530; doi:10.1371/journal.pone.0262153)
Supplement: Attachment — Submitted filename: PLOS One Response to Reviewers.docx [file pone.0262153.s004.docx]

Prof. Ritesh G. Menezes, M.B.B.S., M.D., Diplomate N.B.

Academic Editor, PLOS ONE

Date: December 8th, 2021

Dear Prof. Ritesh G. Menezes,

Thank you for the feedback for our manuscript, "Motivators and barriers to research participation for individuals with cerebral palsy and their families". We appreciate the opportunity to reply to the reviewers and to submit a revised version of our manuscript for further consideration.

The reviewers provided some important insights and critiques of our resubmitted manuscript. We have made relevant changes to the text and have responded thoughtfully to all comments in a point-by-point fashion below with review comments in bold followed by our responses. The page numbers and line numbers referenced in our responses are all from the manuscript with tracked changes.

Thank you for your consideration of this revised version of our manuscript.

Sincerely,

Theresa Sukal-Moulton, PT, DPT, PhD and other authors

Reviewer(s)' **Comments to Authors** and Responses

**(1) Thank you for your responses to the reviewers. I have nothing further to add and look forward to seeing the paper in the literature. We all have a lot to improve on in this space.**

We appreciate your comments and for taking the time to review the manuscript.

**(2) Page 8, line 332-333 can you please add more clarity to this sentence: "In addition to direct payment, compensation for other costs associated with travel should be considered." There is an ethical difference between giving someone an incentive to sign up for a research study (e.g., $50 gift card), and reimbursing their expenses (e.g., travel, parking). What exactly do you mean by "direct payment"? In your paper, please be clear on the use of words that are meant to describe a recruitment incentive and expense compensation.**

Thank you for your comments. We intended “direct payment” to be defined as the gift card or check that participants receive as incentive for study participation, but understand the concern that you’re sharing. Therefore, we have edited the sentence per your suggestion to: “In addition to financial incentives for participation, compensation for other expenses associated with travel should be considered” (pg. 18, lines 340-341 in the manuscript with tracked changes).

**(3) First, I would like to thank the authors for their patience during my review of this manuscript. I personally thank the authors for conducting this thoughtful survey - despite the pandemic setbacks. I recommend this encouraging paper for acceptance to PLOS ONE. Recognition of the obstacles that caregivers and individuals with CP face when being included in research is a necessary addition to the literature and empowerment of the disability community.**

Thank you for your kind comments and for taking the time to review the manuscript.

**(4.1) In general, there is a narrative of "indirect benefit" vs "direct benefit" in the Travel Needs and Preferences category under Part III of Methods section. From my understanding, research does not directly benefit those who participate (that would be more of a QI initiative) but rather in some instances there may be the prospect of benefit. Authors can read more here:** [**https://www.ncbi.nlm.nih.gov/pmc/articles/PMC2945615/**](https://urldefense.com/v3/__https://www.ncbi.nlm.nih.gov/pmc/articles/PMC2945615/__;!!Dq0X2DkFhyF93HkjWTBQKhk!GEtE-GGgDg4Za_uZjoEF1v_Cx5Omc2trEMtVqSJtkUp1wXhv9f_45r92-nfZZS3BQAYKSrZ5NjOKNrH__w$)**. Friedman et al argue about “direct” and “indirect” benefits in the context of research on vulnerable populations and children and try to outline what counts as a potential direct benefit, if any.**

Thank you for this insightful comment. While we agree with the definitions outlined by Friedman et al., our approach differed in the survey design. We have added definitions of the “potential for a direct benefit study” vs. an “indirect benefit study” using the language that was in our survey (pg. 6, lines 148-152 and Table 2 in the manuscript with tracked changes). Our intention when asking these questions was to differentiate between studies such as clinical trials (potential for direct benefit) and basic science investigations (indirect benefit). We agree that we did not control for participants’ understanding and interpretation of the likelihood for direct benefit, which is the rationale for using the same definition for readers. This limitation has been addressed in the discussion section (pg. 18, lines 357-360).

**(4.2) Page 14, line 255: Mean response time is reported for “travel time for indirect benefit study” vs “travel time for direct benefit study”. Again, it’s still not clear if this is the participant's perception of the benefit that they may derive from their participation in the study? Research aims to benefit future patients and often participation is altruistic, where research outcomes can benefit the scientific understanding of CP for future treatment and intervention. There is uncertainty around the interventions/therapies being studied. Your findings regarding the barrier “travel time” in light of “direct” vs “indirect” benefits could possibly help ethics review committees make prospective decisions on whether the research in question offers an appropriate risk-benefit profile, as certain prospects of benefit can be seen as decreasing impact of barriers and maybe even the risk involved to those participating. More studies like this survey are recommended.**

We have provided the definitions of “potential for direct benefit” vs. “indirect benefit” according to the language in the survey (pg. 6, lines 148-152 and Table 2). Our question about travel time was related to convenience rather than risk and is therefore likely less relevant to an ethics committee determination. We agree that how benefits are perceived by potential participants is important and should be studied in the future.

**(4.3) I think a brief explanation on what the authors mean by "indirect benefit study" and "direct benefit study" would be meaningful. Maybe an inherent barrier is education regarding the purpose of research, since "indirect benefit" and "direct benefit" is seen impacting barriers like travel time. Mention of therapeutic misconception (inflating likelihood of benefit from research participation by seeing research as routine care) would be useful. Read here:** [**https://www.ncbi.nlm.nih.gov/pmc/articles/PMC3690536/**](https://urldefense.com/v3/__https://www.ncbi.nlm.nih.gov/pmc/articles/PMC3690536/__;!!Dq0X2DkFhyF93HkjWTBQKhk!GEtE-GGgDg4Za_uZjoEF1v_Cx5Omc2trEMtVqSJtkUp1wXhv9f_45r92-nfZZS3BQAYKSrZ5NjPYurKOzQ$)

As mentioned above, we defined these terms very broadly for participants. We have edited the limitations section of the discussion to acknowledge that we did not address considerations such as therapeutic misconception (pg. 18, lines 357-360). We agree that this is an important topic for investigation in future studies.

**(4.4) Page 18, line 335: Again, is this their perception of benefit?**

Yes, this is the participants’ perception of benefit. We have addressed this by clarifying the phrase in the relevant sentence: “...studies with and without potential for direct benefit” (pg. 18, line 343).

**(5) “Research” is referred to broadly. Maybe in the future it would be useful to understand if the barriers to research are associated with the type of research involvement (e.g., research comparing routine standard of care interventions vs research on pre-approval CP therapeutics).**

Thank you for this comment. We agree that this would be an interesting direction for future exploration.

**(6) Page 18, line 352: This is an excellent point among many. Valuable and well-designed research should be informed by stakeholder and patient perspectives.**

Thank you for this kind comment.

**(7) Limitations are well addressed. Great solutions proposed on CP stakeholder engagement.**

Thank you, we’re glad to see a shift towards this engaged approach occurring in research and hope to accelerate it.

**(8)** **Page 19, line 363: “…true partners in research that aims to benefit *people like* them."**

This sentence has been edited as proposed (pg. 19, lines 378-379).
